# Stage effect of chronic kidney disease in erectile function

**DOI:** 10.1590/S1677-5538.IBJU.2017.0228

**Published:** 2018

**Authors:** Márcio Rodrigues Costa, Viviane Campos Ponciano, Théo Rodrigues Costa, Caio Pereira Gomes, Enio Chaves de Oliveira

**Affiliations:** 1 Escola Médica da Universidade Federal de Goiás, GO Brasil; 2 Mercy Holy House, Goiânia, GO, Brasil; 3 Hospital Geral de Goiânia Doutor Alberto Rassi, GO, Brasil

**Keywords:** Renal Insufficiency, Chronic, Kidney Failure, Chronic, Erectile Dysfunction

## Abstract

**Purpose:**

The study aims to assess the influence of the stage of chronic kidney disease and glomerular filtration rate on prevalence and degree of erectile dysfunction.

**Materials and Methods:**

This transversal study, conducted from May 2013 to December 2015, included patients with chronic kidney disease in conservative treatment, stages III/IV/V. Erectile dysfunction was evaluated by the International Index of Erectile Function. Data classically associated with erectile dysfunction were obtained by medical record review. Erectile dysfunction, degree of erectile dysfunction, and other main variables associated with erectile dysfunction were compared between patients with chronic kidney disease on conservative treatment stages III versus IV/V using the Chi-square test. The relationship between score of the International Index of Erectile Dysfunction and glomerular filtration rate was established by Pearson correlation coefficient.

**Results:**

Two hundred and forty five patients with chronic kidney disease in conservative treatment participated of the study. The prevalence of erectile dysfunction in patients with chronic kidney disease in stages IV/V was greater than in stage III. Glomerular filtration rate positively correlated with score of the International Index of Erectile Dysfunction.

**Conclusions:**

The study suggests that chronic kidney disease progression (glomerular filtration rate decrease and advance in chronic kidney disease stages) worsen erectile function. Hypothetically, diagnosis and treatment of erectile dysfunction may be anticipated with the analysis of chronic kidney disease progression.

## INTRODUCTION

Erectile dysfunction (ED) is a persistent inability to attain and/or maintain an erection sufficient to satisfactory sexual performance (1). This sexual disorder should be promptly investigated and treated because it may impair quality of life and health status of its carriers (1-3). In ED investigation, chronic kidney disease (CKD) is a frequent disorder found as a possible cause, but it can also be a consequence (4, 5). Curiously, ED seems to be forgotten and sometimes neglected in that health condition (6).

Many studies have analyzed ED in patients with CKD. The ED prevalence found in these works was usually high (5-10). Age, depression, diabetes mellitus, cardiovascular diseases, duration of CKD, being married, being employed, and not using some antihypertensive drugs (angiotensin-converting enzyme inhibitors) were factors involved with ED in patients with CKD (5-10).

Some studies specifically evaluated patients with ED and chronic kidney disease on conservative treatment (CKDCT) (9-14). In these patients, ED prevalence found was variable, but usually high. Among risk factors for ED, diabetes mellitus and time of CKD stood out (8). The role of CKD stages and the glomerular filtration rate (GFR) as risk factors on ED aroused attention because no unanimous response was found. Some studies show association of ED with GFR and CKD stages, others only in specific population Groups or do not establish this relationship in any way (8, 10, 11, 15-18). Therefore, our study evaluated the influence of CKD stages and GFR on the ED prevalence and degree to clarify this point.

## MATERIALS AND METHODS

This transversal study, conducted from May 2013 to December 2015, was approved (registration no. 090/2011) by the Ethics Committee of the Federal University of Goiás Clinical Hospital, in accordance with Helsinki Declaration of 1975 revised in 1983.

For practical purposes, CKDCT was defined as structural or functional abnormalities of the kidney, present for more than 3 months, with health implications, in patients who did not receive renal replacement therapy, dialysis, or renal transplantation.

Male patients, volunteers, heterosexuals, carriers of CKDCT, from two hospitals in the city of Goiânia (Brazil), without cognitive or communication impairment and that signed an informed consent form were included in this study. The patients were classified into CKD stages according to the criteria of the Kidney Disease Outcomes Quality Initiative (2012) (19) (Table-1). Some descriptive data of these patients, presented in this work, were submitted in another research (10).

**Table 1:** Chronic Kidney Diseases stages.

Stages	Glomerular filtration Rate (mL/min/m^2^)
I	> 90
II	89-60
III a	59-45
III b	44-30
IV	29-15
V	< 15

In the absence of evidence of kidney damage, stage I and II do not fulfill the criteria for Chronic Kidney Disease.

Patients with creatinine clearance, estimated by the Cockcroft-Gault equation (20), greater than or equal to 60mL/min/1.73m^2^, or without data to estimate it (weight, age, or serum creatinine value), were excluded.

Patients were individually approached, before their scheduled medical visit, in nephrology services, previously cited. They received a brief explanation about the research and were instructed to read and interpret questions about erectile function of the International Index of Erectile Function (IIEF). Life habits, sociodemographic characteristics, clinical and laboratory data were obtained from medical records review made by five researchers.

The variables evaluated were: age, marital status, body mass index, systemic arterial hypertension, diabetes mellitus, coronary artery disease, congestive heart failure, time of CKD, smoking, pack years of smoking, alcohol use, antihypertensive numbers, antidepressants and anxiolytics use, hematocrit, cholesterol, high density lipid cholesterol, low density lipid cholesterol, and triglycerides.

ED was assessed by erectile function domain of the IIEF (questions numbers 1 to 5 and 15) (21). According to answers of the questions, patients obtained scores of 1 to 30. Patients were classified according to their scores as follows: no ED (score 25 to 30); mild ED (score 19-24); mild to moderate ED (score 13 to 18); moderate ED (score 7-12), and severe ED (score 1-6).

Data were tabulated in Microsoft^®^ Excel 2007 and analyzed by Statistical Package for Social Sciences for Windows 16. Descriptive analysis was done for ED and its categories (according to IIEF), life habits, sociodemographic characteristics, and clinical and laboratory data. Categorical variables were assessed by ED proportion in each category and continuous variables by ED proportion in categories created to facilitate interpretation of results. ED prevalence, ED degrees, and each categorical variable of patients with CKDCT stage III were compared with their corresponding stage IV/V, using Chi-square test. Severe ED was compared with other ED degrees in patients in stage III using the previous test. Stage IV/V patients were grouped and similar comparison was done. P equal or less than 0.05 was considered statistically significant. Relationship between IIEF score and GFR was analyzed by Pearson correlation coefficient.

## RESULTS

**Figure 1:**
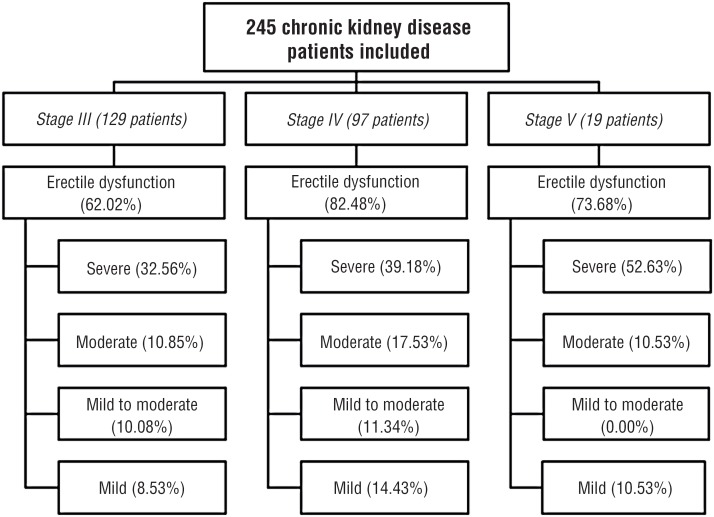
Erectile dysfunction and the proportion of severe degree in each stage.

**Figure 2:**
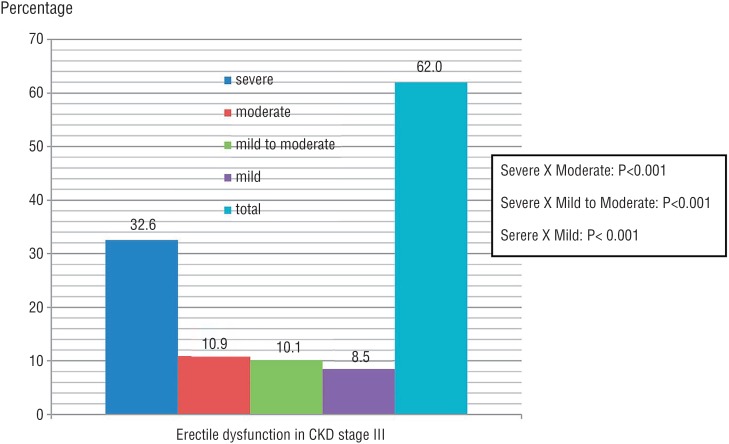
Comparison of severe versus moderate, mild to moderate, and mild erectile dysfunction in patients with CKD on conservative treatment in stage III. Estimated by chi-square test; P=Statistical significance; CKD=Chronic Kidney Disease.

**Figure 3:**
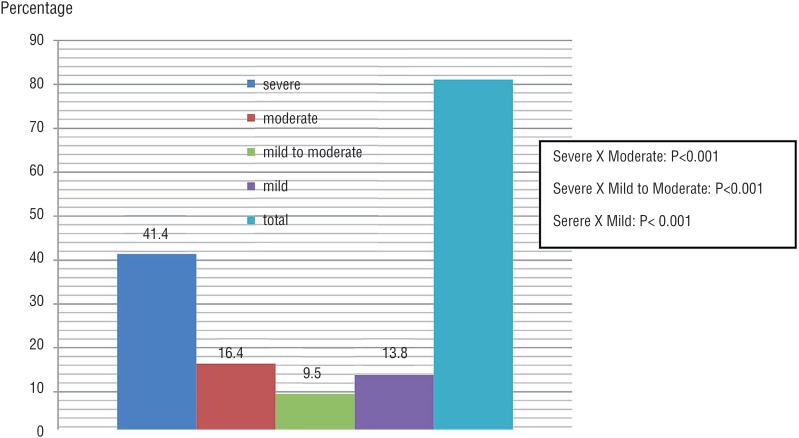
Comparison of severe versus moderate, mild to moderate, and mild erectile dysfunction in patients with CKD on conservative treatment in stage IV/V. Estimated by chi-square test; P=Statistical significance; CKD=Chronic Kidney Disease.

The study assessed 338 patients with CKDCT, 245 (72.49%) patients were included and 93 (27.51%) excluded. Mean age was 63.36±13.74, 67.16±14.43, and 66.52±12.27 years in stages III, IV, and V, respectively. Creatinine clearance (estimated) means were 33.34, 23.07, and 12.14mL/min/1.73m^2^ in same stages, respectively. CKD stages, ED, and severe ED distribution of patients are presented in Figure-1. In patients with CKD stage IV/V, severe ED frequency was higher than in other ED degrees. In patients with CKD stage III, severe ED frequency also showed this behavior (Figures 2 and 3).

Potential factors that can influence erectile function occurred in similar frequency in patients with CKD stage III and IV/V. Only body mass index was not similar between them (Table-2).

**Table 2:** Patients with chronic kidney disease on conservative treatment, stage III versus stage IV/V. Pairing among main variables that could influence erectile function.

Variable	Chronic kidney disease (stage) a				P-values *
	III		IV/V		
	n	%	n	%	
Age ≥ 50 years	109	84.5	101	87.1	0.566
Married status	86	66.7	64	55.2	0.065
Body Mass Index ≥ 25	72	56.3	50	43.5	0.047
Arterial hypertension	107	82.9	99	85.3	0.608
Arterial hypertension ≥ 10 years	61	57.5	51	53.7	0.582
Diabetes	49	38.0	35	30.2	0.198
Diabetes ≥ 10 years	34	72.3	27	79.4	0.466
Coronary artery disease	22	17.3	15	13.3	0.386
Congestive heart failure	14	11.0	13	11.5	0.906
Chronic renal disease ≥ 10 years	18	15.3	23	20.5	0.296
Active smoker b	9	7.0	15	12.9	0.117
Pack-year index ≥ 20	57	65.5	51	64.6	0.897
Current alcohol user c	22	17.1	13	11.2	0.192
Antihypertensive ≥ 4	21	16.3	22	19.0	0.581
Anxiolytic	10	7.8	4	3.4	0.147
Antidepressant	14	10.9	9	7.8	0.407
Hematocrit ≥ 30%	122	95.3	104	89.7	0.091
Cholesterol ≥ 200 mg/dL	29	23.6	30	27.3	0.517
Cholesterol HDL < 40 mg/dL	55	44.4	57	52.8	0.200
Cholesterol LDL ≥ 130 mg/dL	27	22.1	27	25.5	0.554
Triglycerides ≥ 150 mg/dL	50	40.0	41	37.3	0.668

* Estimated by chi-square test; ^a^ Classification according to Kidney Disease Outcomes Quality Initiative; **P** = Statistical significance; ^b^ Patients that smoked more than 100 cigarettes in any time of life and actually smoke; ^c^ Regular use of alcohol.

**Figure 4:**
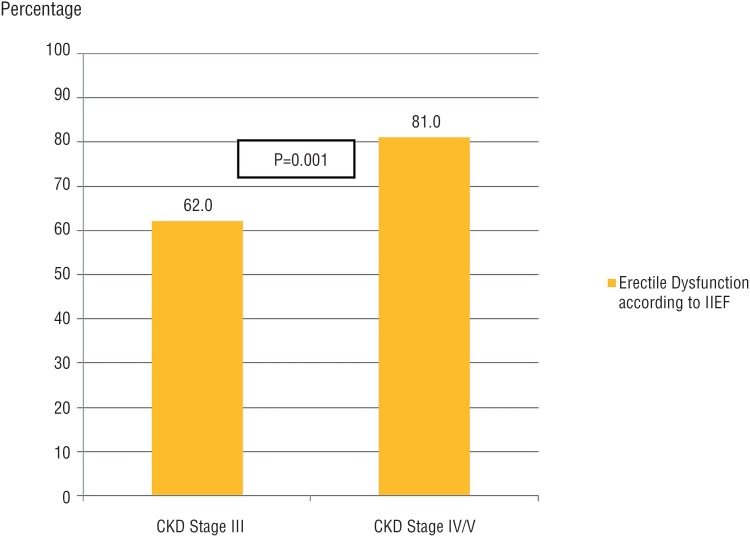
Comparison of erectile dysfunction among patients with chronic kidney disease on conservative treatment in stage III versus IV/V. Estimated by chi-square test; P=Statistical significance; CKD=Chronic Kidney Disease; IIEF=International index of erectile function.

**Figure 5:**
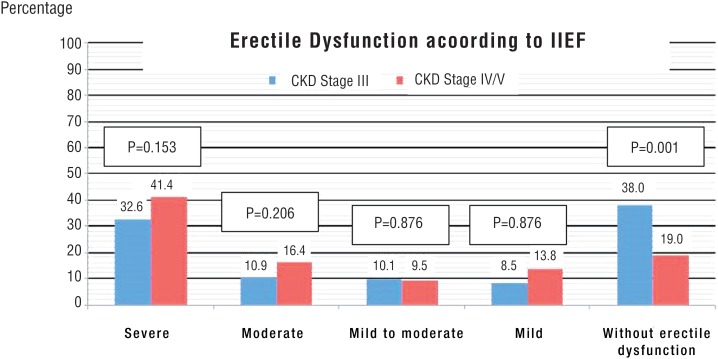
Comparison of category of erectile dysfunction among patients with chronic kidney disease on conservative treatment in stage III versus IV/V. Estimated by chi-square test; **P**=Statistical significance; **CKD**=Chronic Kidney Disease; **IIEF**=International index of erectile function.

ED proportion in patients with CKD stage III was lower than in stage IV/V (Figure-4). However, proportion of severe ED did not differ between these stages (stage III versus stage IV/V). The same also occurred with other ED degrees (mild, mild to moderate, and moderate) (Figure-5).

**Figure 6:**
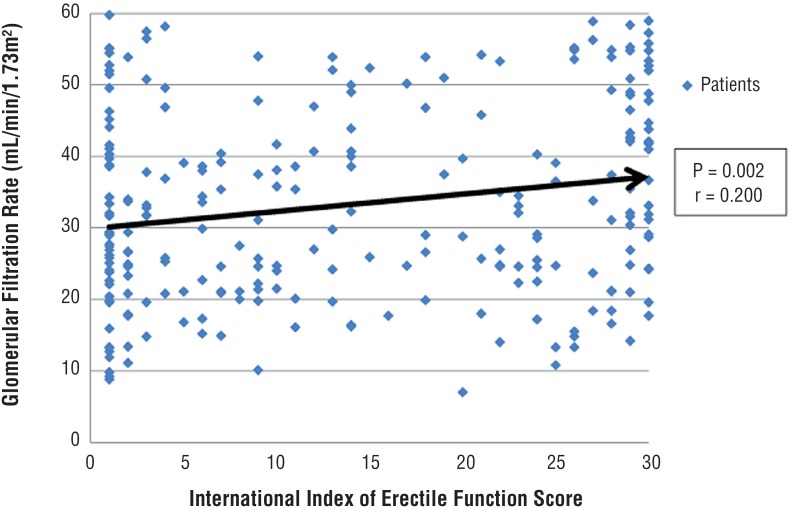
Relationship of International Index of Erectile Function score and glomerular filtration rate. **P**=Statistical significance; **r**=Pearson correlation coefficient.

Increase of IIEF score followed the increase of GFR with a weak relationship (Figure-6).

## DISCUSSION

The first reference of sexual disorder prevalence, in patients with CKDCT, estimated 9% in both sexes (12). Other works have evaluated erectile function in uremic patients under conservative treatment. Usually, the ED prevalence found was higher than that found in the first reference. In addition, research mostly showed severe ED more frequently than other ED classifications (mild, mild to moderate, and moderate ED) (8, 9, 11-14, 22). ED prevalence was 40% in a study of 25 male uremic patients on conservative treatment (13). Research with 53 men in CKD reported erectile disorders in 41.5% of them, before starting hemodialysis (14). One study found ED in about 30% of all uremic patients under conservative treatment (9). ED was observed in 76.5% of 81 outpatients in a study of men with CKDCT stages III, IV and V. ED was mild, mild to moderate, moderate, and severe in 29.6%, 18.5%, 16%, and 12.3% of the patients, respectively (8). ED prevalence was 84% in a research in which 26 patients had estimated GFR between 15 and 90/min/1.73m2 (8). Work that analyzed 15 pre-dialysis men with ED showed severe ED in 20% of them (22). The present study evidenced high ED prevalence in patients with CKDCT, and to make matters worse, more than a half of patients in this work with ED and CKDCT, in stage V, had severe ED.

Some studies registered influence of CKD stages and GFR on erectile function (10, 12, 15, 17, 18, 23). Apparent worsening or worsening of sexual function, with progression of uremia, was reported by 137 (48%) of 287 men on hemodialysis, before starting this treatment (12). Forty-five percent of 32 male married patients on dialysis reported sexual potency decrease, after onset of renal disease (23). Direct correlation between IIEF and GFR, in outpatients with CKD, stages III and IV, was showed in preliminary results (18). Analysis of 183 men undergoing coronary angiography, to detect acute myocardial infarction, found ED correlating with lower GFR, in patients with single arterial coronary disease (15). Patients with type 2 DM had ED associated with albuminuria and lower GFR levels (17). Adult male patients with ED and chest pain, presumably of coronary origin, had a worse GFR (24). As the severity of the ED increased, a more severe decrease in the creatinine clearance occurred in male subjects with chest pain (25). Observational research involving 270 consecutive hypertensive male patients showed a significant correlation between the ED score and GFR (26). Work with a primary aim of investigating risk factors of obstructive sleep apnea demonstrated that patients with ED had lower GFR (27).

Influence of CKD stages and GFR in erectile function is not always found. ED was observed in 72.3%, 81.5%, and 85.7% of 81 outpatients with CKD stages III, IV, and V, respectively. These ED rates were not statistically different (8). Male patients with estimated GFR between 15 and 90mL/ min/1.73m2 (26 patients) were compared with age-matched controls (20 patients) with estimated GFR equal or greater than 90mL/min/1.73m2. ED was found in 84% and 75% of patients, respectively. These different ED rates did not represent statistical difference (11). Patients with type 2 diabetes had ED associated with microangiopathy and diabetic retinopathy, but not with the GFR levels (16). A single-center, prospective cross-sectional study with non-diabetic male patients, stage III and IV CKD did not find ED associated with GFR (28). Male patients from a peritoneal dialysis center had daily urine volume positively associated with ED but not with the GFR (29).

The present study found lower GFR levels and/or more advanced stages of CKD associated with worse erectile function. Some works have shown that worse kidney function increases the chance and severity of sexual disorders (12, 15, 17, 18, 23). Association of CKD and ED could be explained as a consequence of injuries caused directly or indirectly by CKD. CKD can interfere directly with erectile function by decreasing penile arterial blood flow, affecting penile venous leakage due to shunts, altering penile smooth muscle function, and causing hormonal disturbances and/or neurogenic dysfunction (30). Indirectly, CKD impairs erectile function by requiring use of medications with many side effects (including ED) or linked to diabetes mellitus and/or systemic arterial hypertension, conditions that lead to endothelium dysfunction causing CKD and ED (1, 3, 8, 9, 30, 31).

Assuming conditions resulting from CKD that directly cause damage to erectile function are magnified in the more advanced disease, it could also be assumed that the more severe the CKD is, the greater severity and frequency of ED. Another hypothesis is that the more serious, poorly treated, or uncontrolled diabetes and/or hypertension are, the more serious the CKD and ED resulting from them are. Therefore, the studied patients with CKD stages IV/V may had higher ED prevalence than patients with CKD stage III, because the first are more expose to directly deleterious effects of CKD in the penis or could have worse, more poorly treated or uncontrolled diabetes and/or arterial systemic hypertension than patients with CKD stage III. The present work did not evaluate severity and control of diabetes or arterial systemic hypertension, a limitation of the study.

The work has other characteristics that may have affected the results. This is an observational study. Therefore, association among studied variables can be found, but causal relationships cannot be proved among them. Non-random samples were used in this work, thus the samples may have been subject to bias. Evaluation of some variables cited as influential in erectile function (prolactin, testosterone, and zinc levels, etc.) was not performed in the present research (32, 33). These unevaluated variables might not have been equally distributed between patient Groups compared (stage III versus IV/V) in the study. Thus, ED prevalence in the groups could have been altered.

## CONCLUSIONS

In conclusion, this study suggests that CKD progression (GFR decrease and advance in CKD stages) worsen erectile function. Hypothetically, diagnosis and treatment of ED may be anticipated with the analysis of CKD progression. Therefore, activity and sexual health may be preserved or re-assumed by CKD patients, and their quality of life may be improved.

## LIST OF ABBREVIATIONS

ED: = Erectile dysfunction
CKD: = Chronic kidney disease
CKDCT: = Chronic kidney disease on conservative treatment
GFR: = Glomerular filtration rate
IIEF: = International index of erectile function
